# Some Pathological Causes of Dysmenorrhea*Read before the North Texas Medical Association, at Paris, June 17, 1907.

**Published:** 1907-09

**Authors:** Henry K. Leake

**Affiliations:** Dallas, Texas


### TEXAS MEDICAL JOURNAL.

Established July, 1885.

F. E. DANIEL, M. D., - - - - - Editor, Publisher and Proprietor

PUBLISHED MONTHLY.—SUBSCRIPTION $1.00 A YEAR.

VOL. XXIII. AUSTIN, SEPTEMBER, 1907. No. 3.

The publisher is not responsible for the views of contributors.

For Texas Medical Journal.

### Some Pathological Causes of Dysmenorrhea.*

BY HENRY K. LEAKE, A. M., M. D., DALLAS, TEXAS.

Dysmenorrhea signifies difficult menstruation, not painful menstruation, although both conditions are included in the orthodox definition. Menstruation may be scant and difficult, as in some cases of undeveloped uteri, and not painful, while in others, as in membranous dysmenorrhea, the flow may be profuse and not difficult, yet exceedingly painful; further examples could be mentioned. A word might be found to express painful menstruation leaving dysmenorrhea to bear its own burdens, which are numerous enough. However, I accept the text-book definition and to limit the discussion of the portion of the subject allotted to me, prefer to speak of some major pathological causes of dysmenorrhea rather than of its varied pathology, for this would involve an almost endless detail unnecessary to carrying out the practical design of the symposium. Nor shall I rely wholly upon the literature, but attempt an outline of the mental process which develops ordinarily in the course of my examinations of patients who present themselves with dysmenorrhea, often in aggravated and rebellious form.

I assume that the fundamental factor in all varieties of dysmenorrhea is congestion, either normal or abnormal, arterial or venous. It is an essential element of normal menstruation. Without congestion, no menstruation, but ovulation may continue actively. Every twenty-one or twenty-eight days there is recurrent congestion of all the pelvic organs. The ovaries secrete a hormone

*Read before the North Texas Medical Association, at Paris, June 17, 1907.

which, passing into the blood stream, exerts its altruistic stimulation (Campbell) upon the menstrual nerve center whence an impulse is sent chiefly to the pelvic arterial system in which the blood pressure is increased, resulting in rupture of the endometrial capillaries followed by relaxation of the arterial vessels throughout the entire pelvic area. I have employed the words "blood pressure" without positive opinion as to the exact state of the arterial vessels in normal menstruation, whether these are contracted or dilated; for inasmuch as in many patients with dysmenorrhea there is evidence of the former condition of the arteries, not alone in the pelvis, but throughout the body, as dysmenorrhea is not possible without congestion and as the pains are quickly relieved by a vaso-motor dilator, such as nitro-glycerine (Mary Putnam Jacobi), it appears that blood pressure here as has been claimed in conditions of shock may not so much vary with the size of the arteries as we are accustomed to recognize. Otherwise, why, in explaining the nature of the shock which these cases of dysmenorrhea so much resemble, should Malcolm protest their contraction while Crile, Mummery and others assert the contrary? If we believe that in shock a preliminary stage of arterial contraction obtains, so also in this certain type of dysmenorrhea wherein just prior to the establishment of the flow the patient has "facial pallor, blueness of the lips, coldness of the extremities and a sense of pelvic engorgement," symptoms promptly relieved by the administration of a good vaso-motor dilator, preferably nitro-glycerine.

The pelvic arterial circulation is abundant, but the veins comparatively are large, numerous, tortuous and without valves (Montgomery contra); the mutual adjustment of the two systems being delicate, and, as it were, set upon a hair trigger which may be sprung by the slightest disturbing causes. Contraction of the arteries combines with a sluggish venous current to produce painful engorgement. Such causes may be cold, fright, anxiety, shock and so forth; a weak and irritable nervous system, congenital or acquired predisposing by its inherent instability, as may be seen in girls at boarding schools or those having sedentary occupations, particularly if deprived of air and sunlight. The normal menstrual congestion—menstruation—now becomes pathological—dysmenorrhea—and when established as it may be in a more or less chronic form, constitutes a special variety of the disease rightly named congestive dysmenorrhea. Under this head also some authors have placed gout and rheumatism, well known causes of sluggish circulation in the abdominal organs and a faulty metabolism which finds expression in the pelvic area during the normal activity of the organs concerned in menstruation.

Menstrual congestion super-added to the liberal blood supply developed in the growth of pelvic neoplasms favors congestive dysmenorrhea. Uterine and broad ligament fibroids, especially when impacted in the pelvic basin, are examples of this class. The several displacements of the uterus, notably the retroversio flexed organ are familiar instances. Pressure upon the thin-walled veins obstructs the return circulation, entailing engorgement and pelvic pain at the menstrual period. In the pathology of some writers prolapsed ovaries assume so great importance in dysmenorrhea as to justify their frequent removal. In my opinion this deduction is unwarranted. If displaced, enlarged and adherent their removal may be justified, but this is done for inflammation, not for displacement, with attendant congestion. According to Eli Van De Walker, a well known authority on displacements, "The position of the ovaries in the pelvis is a minor matter and unattended by either functional or sensory symptoms; these are due to other pelvic conditions."

Well-developed ovaries may coexist with undeveloped uterus which affords a limited surface for menstruation, the amount of undergrowth varying in different cases, hence difficult and painful flow. I recognize two types: That wherein there is no local nor constitutional disturbance, and another marked by severe dysmenorrhea; the difference owing to the inherent nervous stamina possessed by the former. If treatment avails to perfect development, as claimed by Carstens, by the use of intra-uterine stems, the dysmenorrhea and its accompanying sterility are cured. Lawson Tait made much of this form of dysmenorrhea for which he promptly operated by removing the adnexæ; I suspect that his diagnosis ofttimes was intuitive, not logical.

Exceptionally the menstrual flow in congestive dysmenorrhea is copious and attended by small clots throughout the period. This may be witnessed during the first months of menstrual life in some subjects where the endometrial arteries are undeveloped in their muscular coats and easily rupture, or be owing to an harmonic stimulation of the menstrual center disproportionate to the needs of the function, but which probably in time is regulated without resort to the curette, although this instrument may be required finally, especially in the former class of patients.

I attempt not to mention all the existing causes which may derange the equilibrium of the pelvic circulation and so cause congestive dysmenorrhea without palpable structural lesions in the pelvis. Such a disturbance is met with most frequently in nulliparous women, but in these and in the parous, while congestion is the primal factor in the causation of pain, the *vis resistentiæ*, both of the local and general nervous systems, is essential to its presence, quality and permanence. Therefore, the severity of congestive dysmenorrhea varies with the nervous stability of the individual in whom it occurs—an important point in practice which should not be overlooked.

But the normal harmonic congestion of the pelvis may be augmented by structural change in the pelvic tissues themselves, with or without pain, and so merging, constitute a variety of disease—congestive—inflammatory dysmenorrhea. It is in this field alone, perhaps, where the abdominal operator for dysmenorrhea may find a safe retreat from the shafts of adverse criticism, for in these circumstances it is often required to ablate the diseased structures, or by conservative operations, such as breaking up adhesions and restoring the positions of displaced organs where, by readjusting the circulation, allows them to functionate with painless regularity.

Chronic inflammation, latent or pronounced, at any point in the pelvis, is aggravated by menstrual congestion which may superinduce a so-called recrudescence, as witness adnexal inflammation, chronic metritis, chronic endometritis, or chronic peritonitis with its varied origin and character. As a frequent cause for dysmenorrhea so-called ovaritis is emphasized in modern pathology. Ovaries knobbed, serrated and hardened, large or small ("cirrhotic"), with or without adhesions associated with painful menstruation are assumed to be its cause; chronic inflammation, the pathological factor. This is doubtful. Such ovaries are discovered often when no dysmenorrhea is present; adhesions are the crucial test. What is the real pathology of these cases? Coe and others assert inflammation. Is the pathology parallel with certain disease in the liver and kidneys? If so the symptomatology is unlike. Removal to cure the dysmenorrhea may succeed for menstruation stops; the pathological cause is not demonstrated. The argument is much the same for so-called "cystic ovaries"—small cysts seldom larger than a buckshot embedded in the stroma or projecting from the surface of the ovary. Upon the altar of this startling phrase ovaries are still sacrificed—a practice which represents often superficial examination and pathological error. The ovary may be loose, but the cysts are said to be the end-product of inflammation, possibly septic; their exact origin, however, is problematical. Ovarian neuralgia—menstrual and intermenstrual—has been believed to have its typical expression in "cystic ovaries," which are removed with no certainty of benefit resulting. In the sense implied, if ovaries are cystic or altered by parenchymatous inflammation, unless adherent, they are harmless. "The mobility of these organs is their factor of safety." (Van De Walker Herman.) Coe says, "The same ovaries 'cystic' which were formerly removed, were punctured, burned, resected and otherwise tampered with (as we now know), they had better been left alone." "Cystic ovaries," as well as the "battered, wrinkled appearing organs" (Van De Walker), large or small, and non-adherent may be pathological, but they cause no symptoms, and a respect paid to them as causes for pain is a salaam to the "fetich of the ovary," which is losing its hold upon the reverence of the pathologist. To relieve pelvic pain the experienced gynecologist will have little to do with the extirpation of "cystic" or "chronically inflamed" ovaries free from adhesions. However, I do not admit that they never demand removal, but base this opinion not upon the pathological changes referred to.

Obstructive dysmenorrhea sometimes is "a condition, not a theory," but commonly a theory unsupported and overworked. I have observed patients with obstinate stenosis of the cervical canal, the result of unskillful amputation of the cervix, sloughing after labor, the use of escharotics without previous dilatation and rough usage by instruments. A lady was operated upon at Strassburg, Germany, for disease of the cervix by circular amputation followed by tortuous contraction of the remainder of the canal and for the relief of which incision and periodical dilatation was necessary until the menopause. Another patient presented a so-called os externum, hidden in a deep recess surrounded by dense exudate, reaching out into the base of the broad ligament, which represented the original tear. She declined operation. A pinhole os is the bugbear of some physicians, but I have many times seen the menstrual blood trickling through it in patients without dysmenorrhea. A lady of good physique complained of sterility and dysmenorrhea. Examination showed a pinhole os and undersized uterus. I prophesied sterility and, unless the adnexæ were removed, continued dysmenorrhea. Several years afterwards she returned and exhibited a lusty boy baby to discredit my pathological acumen. I believe that her dysmenorrhea had vanished. I know little of the stenosis of the isthmus and still less of that of the os internum. The point of the sound may engage in a fold of the mucous lining, or be resented by a sensitive internal os, or the cervix may be flexed forwards and thus mislead the examiner where gentle skill and perseverance might overcome the apparent stenosis.

My views of pathological ante-flexion as a cause of obstructive dysmenorrhea were altered by the researches of Vedeler and Herman. These observers made a large number of examinations in menstruating women with dysmenorrhea and who presented the exact type of ante-flexion believed to obstruct the flow and thus cause dysmenorrhea. In the majority no angularity of the canal, no cohesion of its walls were discovered; the remainder were explained on other grounds. For the posterior displacement the same pathology offered. Moreover, in these cases no hypertrophy of the uterine wall was discovered as exists in the wall of the bladder behind stricture of the urethra. The teaching of Mary Putnam Jacobi is opposed. She claimed that hypertrophy which finally passed into chronic metritis—menstrual metritis—did occur. I recognize this finding also in the dysmenorrhea of single women, but explain its cause upon the theory not of congenital narrowing of the cervical canal, but uterine spasm to overcome a different form of obstruction altogether. This contention may be hair-splitting, but material to the treatment of the disease. Therefore, I question the orthodox teaching of pathological ante-flexion, the extreme flexion of the fundus forward, the cervix pointing normally, causing dysmenorrhea; but the case is different with the cervix curving forward, sometimes almost in a semi-circle. This is a true malformation which I have found often to account for obstructive dysmenorrhea, and may be met with in normal development and position of the fundus as well as in retroversion of an undersized uterus. Endometritis may be engrafted upon this condition to increase further the suffering of the patient.

Obstructive dysmenorrhea may find its cause in the blocking of the cervical canal by fibroids, or a polypus either within the uterine cavity or the cervical canal at the menstrual period acting as a ball valve to produce obstruction.

In some women who suffer from painful menstruation the usual form is spasmodic dysmenorrhea. It has an obstructive feature which confounds it with the disease just mentioned. I accept the theory of its pathology as given by Herman: "There is a property of the uterus which is called 'polarity.' This means that there is an antagonism between the body and the cervix uteri. This is seen during labor; weak pains and an unyielding cervix go together; when the cervix is dilated pains become regular and strong. After delivery in hourglass contraction (or 'relaxation,' as Matthews Duncan more correctly calls it), the cervix uteri is contracted and the body relaxed. When the cervix is dilated the body contracts. In spasmodic dysmenorrhea it is believed that the want of physiological relaxation of the cervix prevents normal regular painless contraction of the body. When the circular fibres of the cervix are temporarily paralyzed by stretching, then the body contracts in a normal way." Organic closure of the os internum is simulated by abnormal sensitiveness at this point which exists in spasmodic dysmenorrhea. "As the disease is not fatal the state of the os internum has never been examined anatomically and, therefore, we do not know why it is sensitive."

This hyperesthesia of the os internum may find its analogue in a similar condition affecting the vesical neck, the polyrus, esophagus, and other regions which require cutting or dilating operations for their relief. It is established as a permanent condition, perhaps, by oft recurring menstrual congestion aggravating an abnormal metabolism existing in the nerve-end organs surrounding the os and the origin of which we can not trace.

In my experience membranous dysmenorrhea is a rare disease, doubtless owing to my failure to diagnose it. Briefly stated: In normal menstruation the endometrial capillaries rupture with molecular shedding of the glandular and surface epithelium; in membranous dysmenorrhea the entire glandular area with its supplying blood vessels, lymphatics and nerves is expelled somatically, or in two triangular or smaller pieces. These block the cervical canal and provoke spasm of the uterus; but they may be passed without pain or, as I have several times observed, the two occurrences may alternate. The pathological factor has not been discovered; theories abound with no substratum of evidence.

Following the lead of Reamy, who published a paper on the subject, I adopted his view that an extraordinarily rebellious form of endometritis is the underlying process which loosens and exfoliates the inner lining of the uterus. Herman regards it as an easily curable affection, and states that it often spontaneously disappears. I can offer no disproof of this; my cases have been tedious and obstinate. This disease may be likened to membranous colitis, proctitis and cystitis, where more or less perfect casts, uplifted by an inflammatory process, may be passed, and, in the case of the bladder, as I have known, with agonizing pain.

I have discussed imperfectly several varieties of an old classification of dysmenorrhea. They are entitled to distinctive emphasis based on critical experience. Ovarian menstrual pain is a pronounced element of congestive and congestive-inflammatory dysmenorrhea. If the pain of a neurasthenic ovary is intensified by the menstrual flow this forms part of the symptom-complex of neurasthenia, and is so classified. "Primary menstrual pain" is considered to be ovarian. It commonly begins with menstruation and ceases about the middle of menstrual life. I have attempted to identify this special form of dysmenorrhea; the result so far is doubtful, but I employ it as a pleasing formula in the case of the unmarried where I am averse to local examination. A clinical history and examination of some married women justify the belief in such a condition of primary menstrual pain originating in the ovary without structural change unless probably it be that asserted by Mary Putnam Jacobi to result from the formation of "anomalous menstrual bodies" disseminated throughout the ovarian stroma—a pathological cause for dysmenorrhea or other pain in the ovary which has not been proved by contemporaneous authority.

Neuralgic dysmenorrhea is almost banal, but women through heredity suffer from migraine and tri-facial neuralgia, why not similarly from neuralgia of the pelvic nerves whose hyperesthetic neurons react unduly to the menstrual congestion? No tissue change may be revealed. The pain selects no special organ in the pelvis; it is universal. However, the classification of neuralgic dysmenorrhea is open to challenge; painful nerves are an element in all forms of dysmenorrhea. Anemia invites the acquired form of neuralgic dysmenorrhea, which is the cry of the nerves for healthy blood.

I have invaded somewhat the domain of treatment, but good pathology often waits on treatment and good health on both.

My colleague will show the full benefits of treatment by discussing this in greater detail.

---

Dr. Leake's Private Hospital at Dallas is being enlarged by fine brick addition, a complete system of hot water heating is being installed throughout the entire hospital, and in many other respects the institution will be improved within the next few weeks. This hospital now has an enviable record and Dr. Leake is to be congratulated upon his continued efforts to make it second to no private hospital in the State.

